# Commentary: Folk-Economic Beliefs: An Evolutionary Cognitive Model

**DOI:** 10.3389/fpsyg.2018.01120

**Published:** 2018-06-27

**Authors:** Tobias Otterbring, Panagiotis Mitkidis

**Affiliations:** ^1^Department of Management/MAPP, Aarhus University, Aarhus, Denmark; ^2^Department of Psychology/CTF, Service Research Center, Karlstad University, Karlstad, Sweden; ^3^Department of Management, Aarhus University, Aarhus, Denmark; ^4^Center for Advanced Hindsight, Duke University, Durham, NC, United States

**Keywords:** lay beliefs, common sense, biases, behavioral economics, evolutionary psychology

More than half a century of research has documented that people's capacity to predict the actions, attitudes, and abilities of themselves and others is quite limited (e.g., Milgram, 1963; Nisbett and Wilson, 1977; Ariely and Loewenstein, 2006; Otterbring et al., 2018). Thus, although scholars have argued that people's common sense and their roles as naïve or intuitive psychologists (Heider, 1958; Ross, 1977) are great assets for theory building, several studies have shown that lay beliefs and other intuition-based predictions “are exaggerated at best, and wholly inaccurate at worst” (Kelley, 1992, p. 6). However, despite findings that lay beliefs, self-report measures, and individuals' intuitions are both unreliable and self-contradictory, and that common sense is an inherently dangerous resource for scholars to rely upon (Fletcher, 1984), there has been a call for more research on the distinction between beliefs and behaviors, between common-sense psychology and scientific psychology (Kelley, 1992).

In a recent paper, Boyer and Petersen (2017) attempt to demonstrate the importance and theoretical interest of lay beliefs, and call for further studies pertaining to this particular topic. While we agree with most of these authors' arguments, we would like to raise some critical points regarding: (1) Their “one-or-none” treatment of alternative accounts for the existence of laypeople's beliefs (based on ignorance, self-interest, and biases), and, more importantly, (2) Their claim that biases primarily constitute “proximate” *how*-explanations, as opposed to “ultimate” *why*-explanations, of various lay beliefs.

Firstly, we are not fully convinced with the way Boyer and Petersen (2017) address the alternative accounts of ignorance, self-interest, and biases. Specifically, we question that lay beliefs exist solely because of one of these sources, mainly because explanations based on ignorance or self-interest are not necessarily at odds with or different from explanations based on biases. In fact, many biases have ignorance (e.g., the overconfidence effect or the Dunning–Kruger effect) and/or self-interest (e.g., the self-serving bias) as their central common denominator (Ross et al., 1977; Bradley, 1978; Weinstein, 1980; Prentice and Miller, 1993; Babcock and Loewenstein, 1997; Lambert et al., 2003).

Taking that into consideration, we also disagree with the thesis that biases typically constitute only proximate explanations of laypeople's beliefs. In our view, a bias account can very well offer ultimate explanations of various lay beliefs. Consider the following examples. Researchers rate their own manuscripts as better than those authored by their peers; in fact, they even believe that their rejected manuscripts are at least as good as others' accepted manuscripts (Van Lange, 1999). Moreover, the majority of people think that they have better sex lives than average (de Jong and Reis, 2015), and that they are more intelligent, competent, and talented than most others (Dunning et al., 2004), especially if they score in the bottom quartile on tests measuring these abilities (Kruger and Dunning, 1999). A plausible ultimate explanation for these “better-than-average”-effects, as well as other self-view-bolstering biases (such as the optimism bias, the overconfidence effect, the egocentric bias, and the self-serving bias), is that it should have been adaptive for individuals, throughout human history, to feel good rather than bad about themselves. Hence, because people tend to portray themselves positively to defend, maintain, and enhance a favorable self-view (Cialdini et al., 1976; Greenwald, 1980; Greenberg and Pyszczynski, 1985), and since cognitive simplification mechanisms make it easier to accept information that confirms prior beliefs (Lord et al., 1979; Nüssler et al., 2018), the evolution of such ego-boosting biases most likely prevented our ancestors from developing a dehumanized dystopia, characterized by a pandemic state of depression. Instead, such biases presumably fostered a climate where we could thrive and survive (cf. Haselton and Nettle, 2006).

Rather than treating biases as idiosyncratic deviations, it may be more meaningful to perceive them as beliefs or behaviors that evolved because they were “good enough” responses providing us with fast answers to specific situations (Goldstein and Gigerenzer, 2002; Todd and Gigerenzer, 2007), particularly those linked to survival and reproduction (Haselton and Nettle, 2006; Haselton et al., 2009). This conceptualization could offer a more unified way to explain the existence of various bias-based beliefs, and we therefore welcome the call for further scientific studies on this topic (Marshall et al., 2013) to elucidate the deep-rooted adaptive aspects of such beliefs.

## Author contributions

TO lead-authored the article, with input from PM. Both authors approved the final version of the article prior to submission.

### Conflict of interest statement

The authors declare that the research was conducted in the absence of any commercial or financial relationships that could be construed as a potential conflict of interest.
